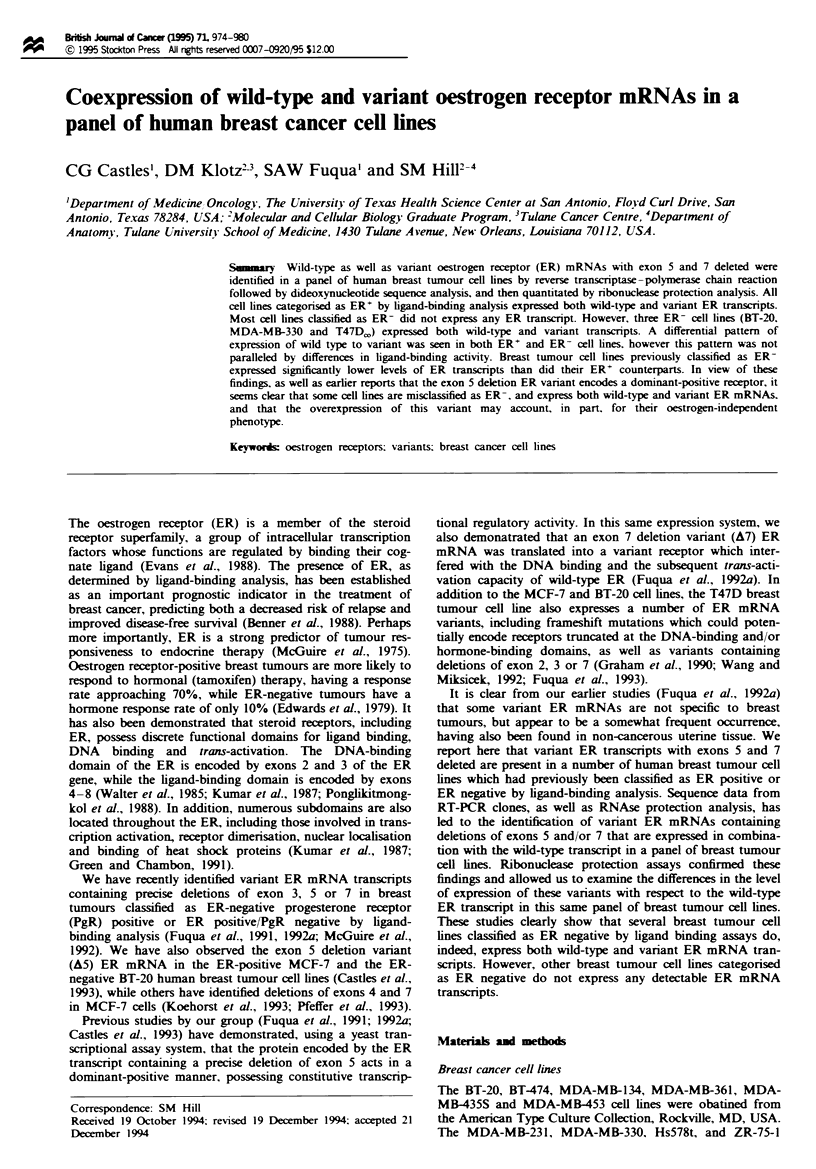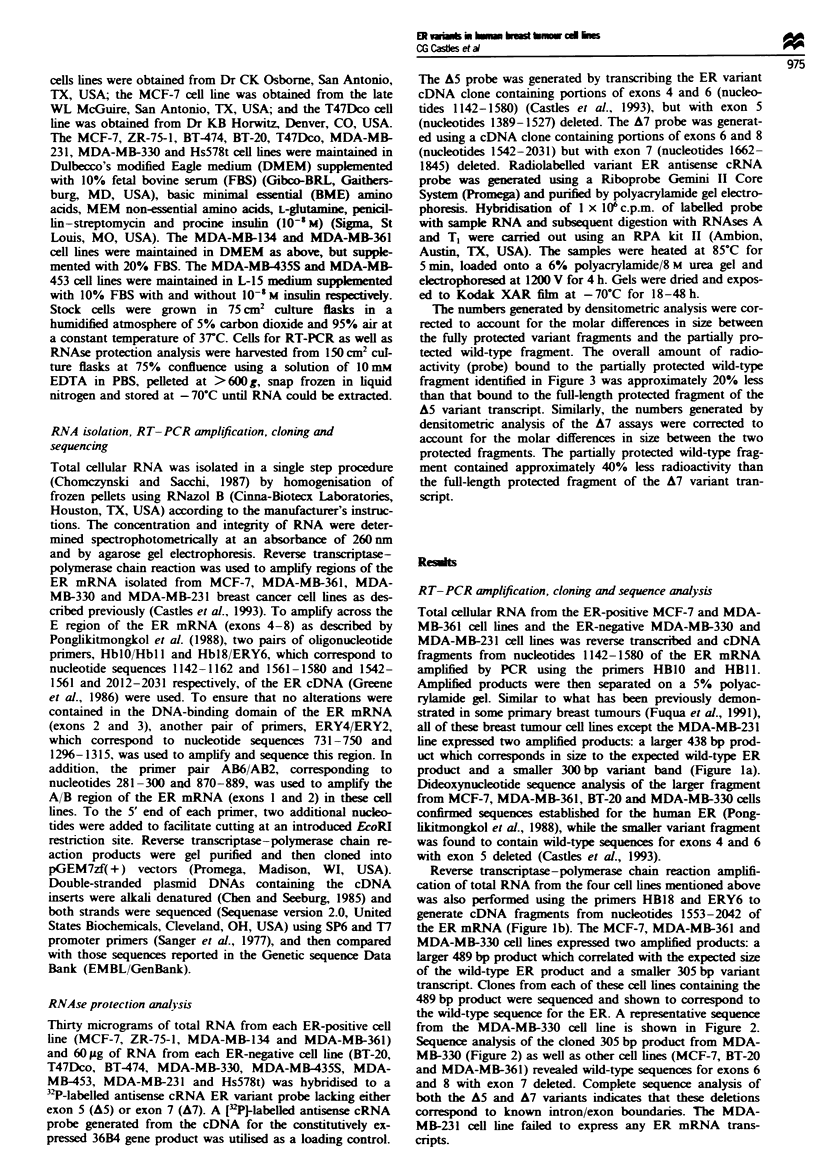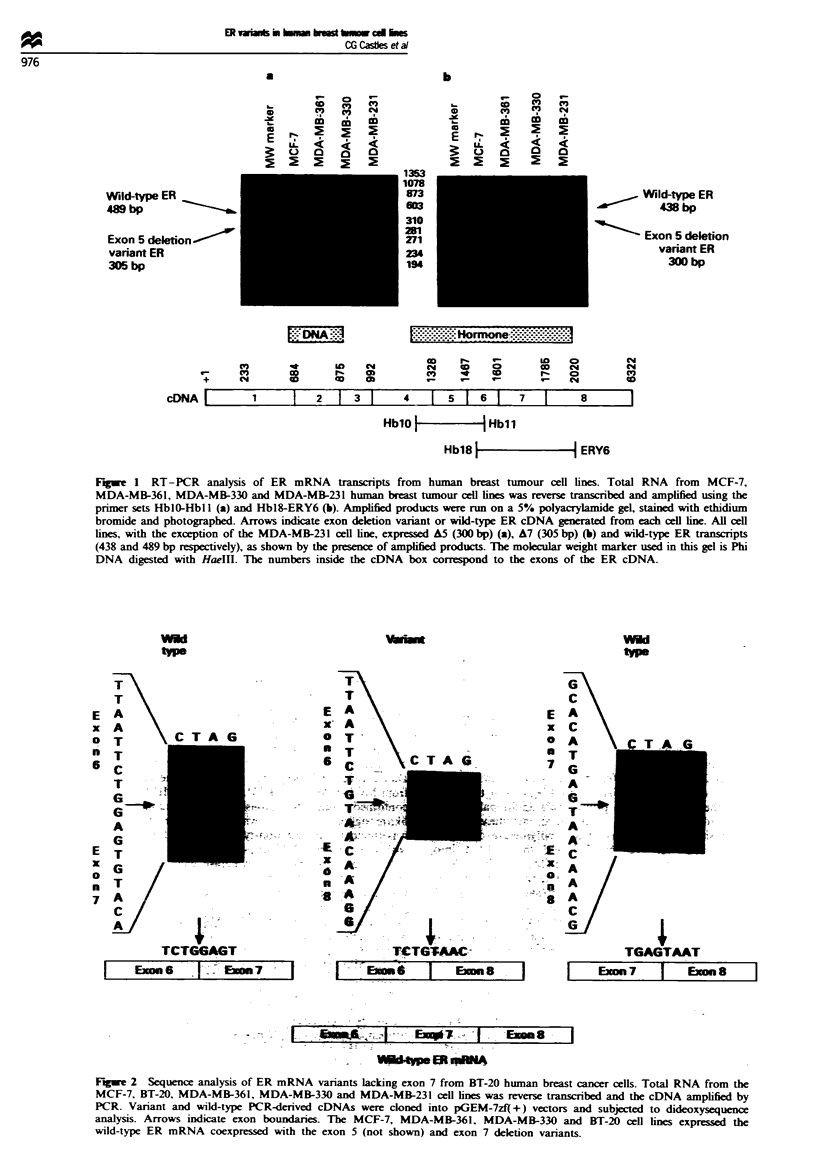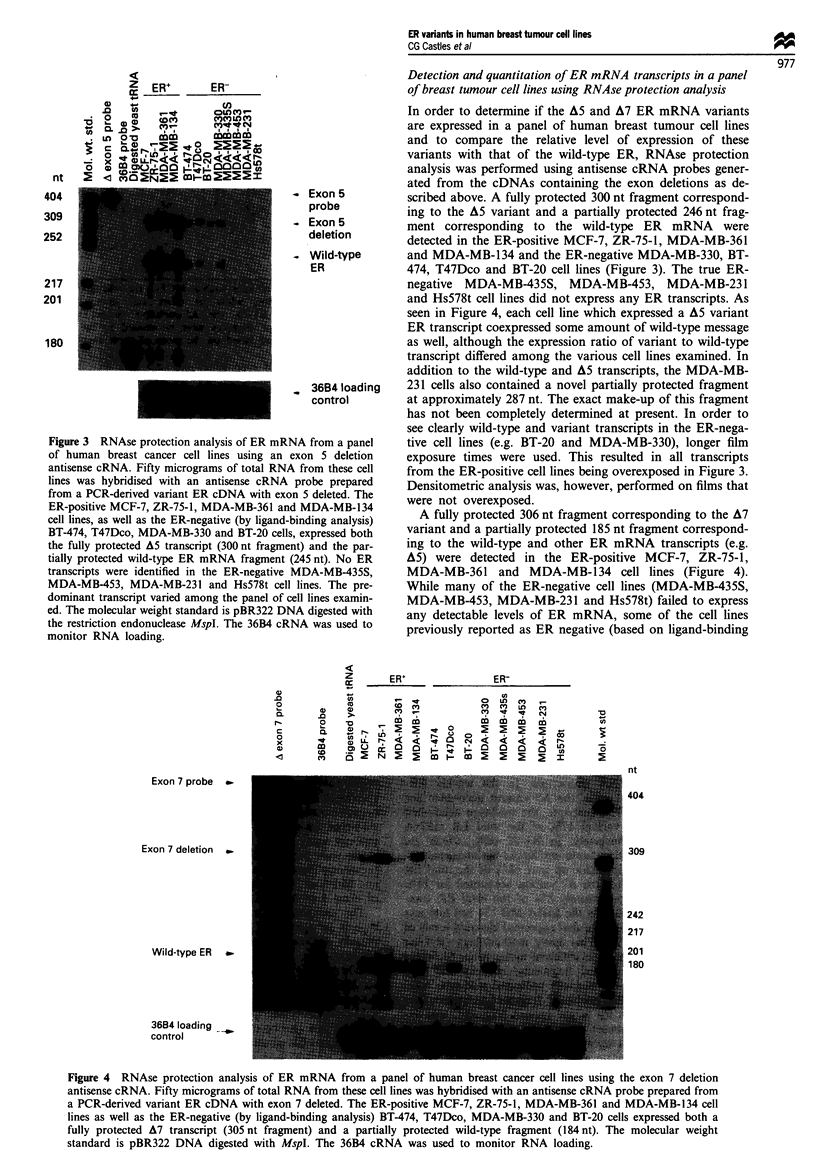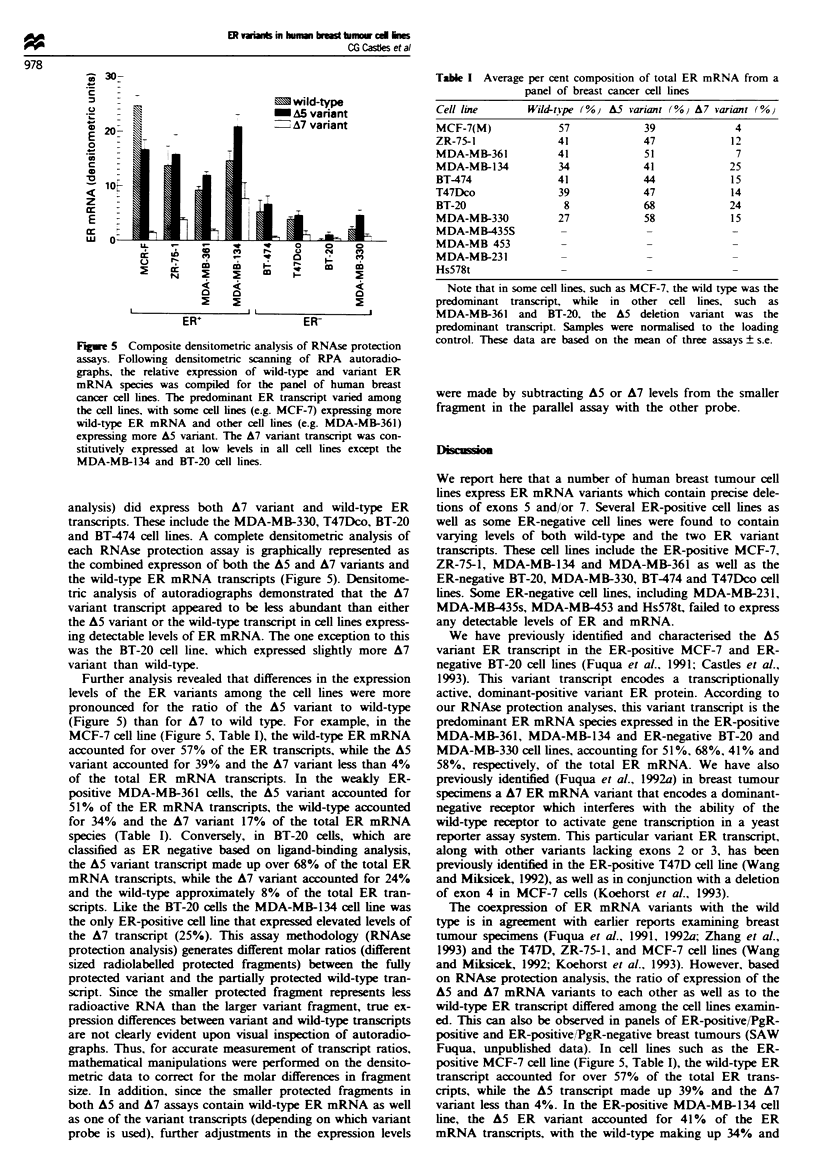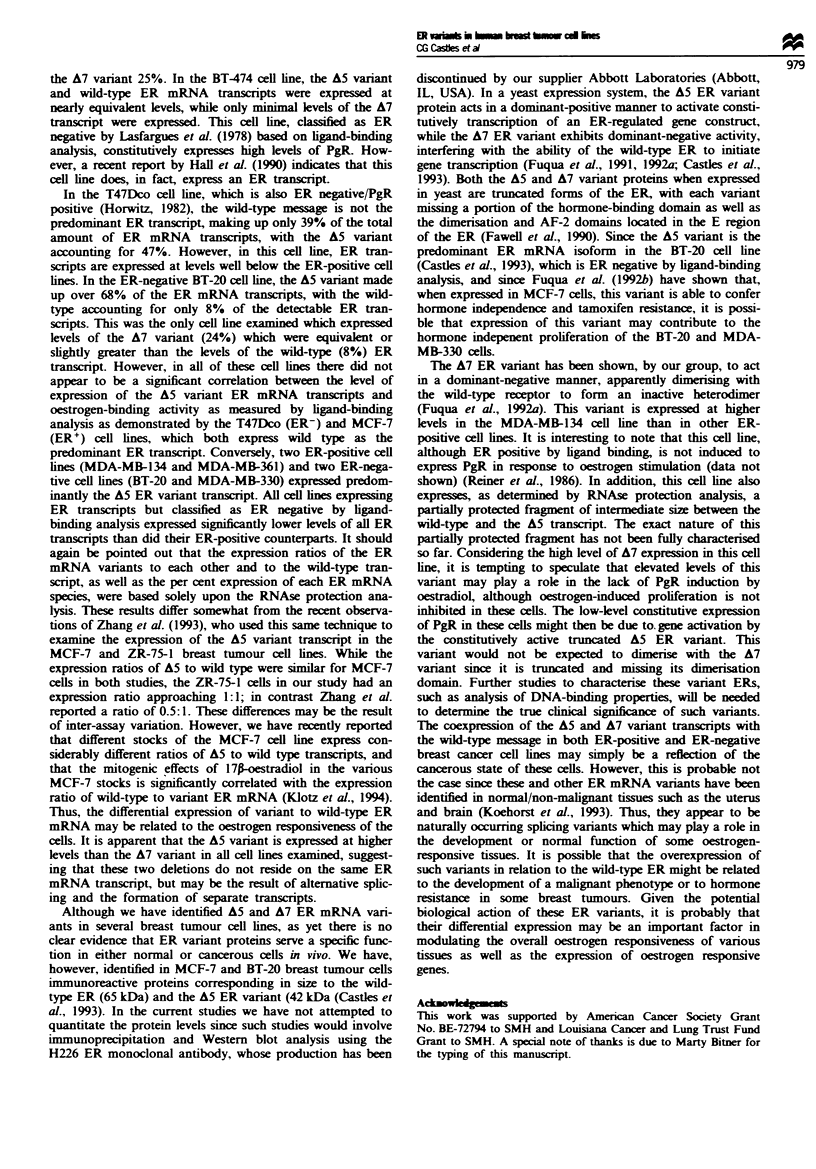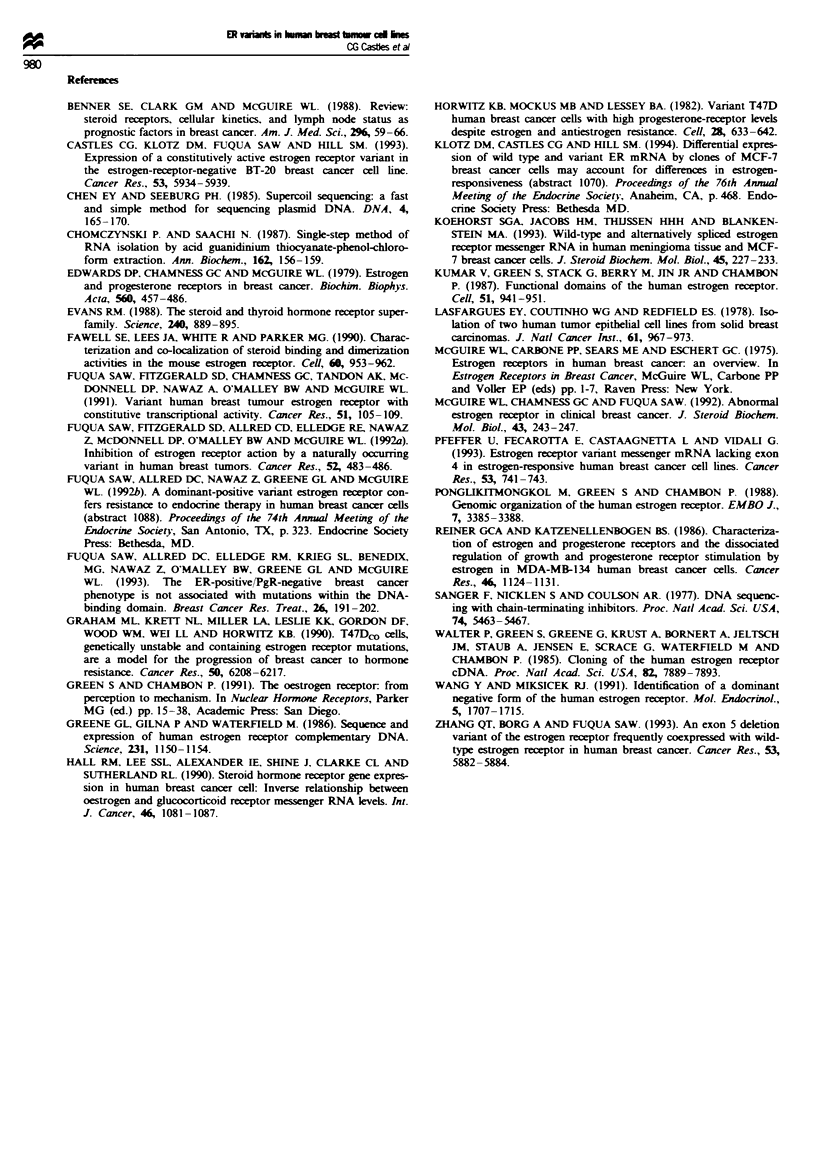# Coexpression of wild-type and variant oestrogen receptor mRNAs in a panel of human breast cancer cell lines.

**DOI:** 10.1038/bjc.1995.188

**Published:** 1995-05

**Authors:** C. G. Castles, D. M. Klotz, S. A. Fuqua, S. M. Hill

**Affiliations:** Department of Medicine/Oncology, University of Texas Health Science Center at San Antonio 78284, USA.

## Abstract

**Images:**


British Journal of Cancer (1995) 71, 974–980

© 1995 Stockton Press All rights reserved 0007-0920/95 $12.00

### Coexpression of wild-type and variant oestrogen receptor mRNAs in a panel of human breast cancer cell lines

CG Castles[1], DM Klotz[2,3], SAW Fuqua[1] and SM Hill[2-4]

[1]*Department of Medicine/Oncology, The University of Texas Health Science Center at San Antonio, Floyd Curl Drive, San Antonio, Texas 78284, USA;* [2]*Molecular and Cellular Biology Graduate Program,* [3]*Tulane Cancer Centre,* [4]*Department of Anatomy, Tulane University School of Medicine, 1430 Tulane Avenue, New Orleans, Louisiana 70112, USA.*

**Summary** Wild-type as well as variant oestrogen receptor (ER) mRNAs with exon 5 and 7 deleted were identified in a panel of human breast tumour cell lines by reverse transcriptase-polymerase chain reaction followed by dideoxynucleotide sequence analysis, and then quantitated by ribonuclease protection analysis. All cell lines categorised as $ER^+$ by ligand-binding analysis expressed both wild-type and variant ER transcripts. Most cell lines classified as $ER^-$ did not express any ER transcript. However, three $ER^-$ cell lines (BT-20, MDA-MB-330 and $T47D_{\infty}$) expressed both wild-type and variant transcripts. A differential pattern of expression of wild type to variant was seen in both $ER^+$ and $ER^-$ cell lines, however this pattern was not paralleled by differences in ligand-binding activity. Breast tumour cell lines previously classified as $ER^-$ expressed significantly lower levels of ER transcripts than did their $ER^+$ counterparts. In view of these findings, as well as earlier reports that the exon 5 deletion ER variant encodes a dominant-positive receptor, it seems clear that some cell lines are misclassified as $ER^-$, and express both wild-type and variant ER mRNAs, and that the overexpression of this variant may account, in part, for their oestrogen-independent phenotype.

**Keywords:** oestrogen receptors; variants; breast cancer cell lines

The oestrogen receptor (ER) is a member of the steroid receptor superfamily, a group of intracellular transcription factors whose functions are regulated by binding their cognate ligand (Evans *et al*., 1988). The presence of ER, as determined by ligand-binding analysis, has been established as an important prognostic indicator in the treatment of breast cancer, predicting both a decreased risk of relapse and improved disease-free survival (Benner *et al*., 1988). Perhaps more importantly, ER is a strong predictor of tumour responsiveness to endocrine therapy (McGuire *et al*., 1975). Oestrogen receptor-positive breast tumours are more likely to respond to hormonal (tamoxifen) therapy, having a response rate approaching 70%, while ER-negative tumours have a hormone response rate of only 10% (Edwards *et al*., 1979). It has also been demonstrated that steroid receptors, including ER, possess discrete functional domains for ligand binding, DNA binding and *trans*-activation. The DNA-binding domain of the ER is encoded by exons 2 and 3 of the ER gene, while the ligand-binding domain is encoded by exons 4–8 (Walter *et al*., 1985; Kumar *et al*., 1987; Ponglikitmongkol *et al*., 1988). In addition, numerous subdomains are also located throughout the ER, including those involved in transcription activation, receptor dimerisation, nuclear localisation and binding of heat shock proteins (Kumar *et al*., 1987; Green and Chambon, 1991).

We have recently identified variant ER mRNA transcripts containing precise deletions of exon 3, 5 or 7 in breast tumours classified as ER-negative progesterone receptor (PgR) positive or ER positive/PgR negative by ligand-binding analysis (Fuqua *et al*., 1991, 1992*a*; McGuire *et al*., 1992). We have also observed the exon 5 deletion variant (Δ5) ER mRNA in the ER-positive MCF-7 and the ER-negative BT-20 human breast tumour cell lines (Castles *et al*., 1993), while others have identified deletions of exons 4 and 7 in MCF-7 cells (Koehorst *et al*., 1993; Pfeffer *et al*., 1993).

Previous studies by our group (Fuqua *et al*., 1991; 1992*a*; Castles *et al*., 1993) have demonstrated, using a yeast transcriptional assay system, that the protein encoded by the ER transcript containing a precise deletion of exon 5 acts in a dominant-positive manner, possessing constitutive transcriptional regulatory activity. In this same expression system, we also demonatrated that an exon 7 deletion variant (Δ7) ER mRNA was translated into a variant receptor which interfered with the DNA binding and the subsequent *trans*-activation capacity of wild-type ER (Fuqua *et al*., 1992*a*). In addition to the MCF-7 and BT-20 cell lines, the T47D breast tumour cell line also expresses a number of ER mRNA variants, including frameshift mutations which could potentially encode receptors truncated at the DNA-binding and/or hormone-binding domains, as well as variants containing deletions of exon 2, 3 or 7 (Graham *et al*., 1990; Wang and Miksicek, 1992; Fuqua *et al*., 1993).

It is clear from our earlier studies (Fuqua *et al*., 1992*a*) that some variant ER mRNAs are not specific to breast tumours, but appear to be a somewhat frequent occurrence, having also been found in non-cancerous uterine tissue. We report here that variant ER transcripts with exons 5 and 7 deleted are present in a number of human breast tumour cell lines which had previously been classified as ER positive or ER negative by ligand-binding analysis. Sequence data from RT-PCR clones, as well as RNAse protection analysis, has led to the identification of variant ER mRNAs containing deletions of exons 5 and/or 7 that are expressed in combination with the wild-type transcript in a panel of breast tumour cell lines. Ribonuclease protection assays confirmed these findings and allowed us to examine the differences in the level of expression of these variants with respect to the wild-type ER transcript in this same panel of breast tumour cell lines. These studies clearly show that several breast tumour cell lines classified as ER negative by ligand binding assays do, indeed, express both wild-type and variant ER mRNA transcripts. However, other breast tumour cell lines categorised as ER negative do not express any detectable ER mRNA transcripts.

### Materials and methods

### *Breast cancer cell lines*

The BT-20, BT-474, MDA-MB-134, MDA-MB-361, MDA-MB-435S and MDA-MB-453 cell lines were obatined from the American Type Culture Collection, Rockville, MD, USA. The MDA-MB-231, MDA-MB-330, Hs578t, and ZR-75-1

Correspondence: SM Hill
Received 19 October 1994; revised 19 December 1994; accepted 21 December 1994

cells lines were obtained from Dr CK Osborne, San Antonio, TX, USA; the MCF-7 cell line was obtained from the late WL McGuire, San Antonio, TX, USA; and the T47Dco cell line was obtained from Dr KB Horwitz, Denver, CO, USA. The MCF-7, ZR-75-1, BT-474, BT-20, T47Dco, MDA-MB-231, MDA-MB-330 and Hs578t cell lines were maintained in Dulbecco's modified Eagle medium (DMEM) supplemented with 10% fetal bovine serum (FBS) (Gibco-BRL, Gaithersburg, MD, USA), basic minimal essential (BME) amino acids, MEM non-essential amino acids, L-glutamine, penicillin–streptomycin and procine insulin ($10^{-8}$ M) (Sigma, St Louis, MO, USA). The MDA-MB-134 and MDA-MB-361 cell lines were maintained in DMEM as above, but supplemented with 20% FBS. The MDA-MB-435S and MDA-MB-453 cell lines were maintained in L-15 medium supplemented with 10% FBS with and without $10^{-8}$ M insulin respectively. Stock cells were grown in 75 $cm^2$ culture flasks in a humidified atmosphere of 5% carbon dioxide and 95% air at a constant temperature of 37°C. Cells for RT-PCR as well as RNAse protection analysis were harvested from 150 $cm^2$ culture flasks at 75% confluence using a solution of 10 mM EDTA in PBS, pelleted at $>600\,g$, snap frozen in liquid nitrogen and stored at −70°C until RNA could be extracted.

### *RNA isolation, RT–PCR amplification, cloning and sequencing*

Total cellular RNA was isolated in a single step procedure (Chomczynski and Sacchi, 1987) by homogenisation of frozen pellets using RNazol B (Cinna-Biotecx Laboratories, Houston, TX, USA) according to the manufacturer's instructions. The concentration and integrity of RNA were determined spectrophotometrically at an absorbance of 260 nm and by agarose gel electrophoresis. Reverse transcriptase–polymerase chain reaction was used to amplify regions of the ER mRNA isolated from MCF-7, MDA-MB-361, MDA-MB-330 and MDA-MB-231 breast cancer cell lines as described previously (Castles *et al.*, 1993). To amplify across the E region of the ER mRNA (exons 4–8) as described by Ponglikitmongkol *et al.* (1988), two pairs of oligonucleotide primers, Hb10/Hb11 and Hb18/ERY6, which correspond to nucleotide sequences 1142–1162 and 1561–1580 and 1542–1561 and 2012–2031 respectively, of the ER cDNA (Greene *et al.*, 1986) were used. To ensure that no alterations were contained in the DNA-binding domain of the ER mRNA (exons 2 and 3), another pair of primers, ERY4/ERY2, which correspond to nucleotide sequences 731–750 and 1296–1315, was used to amplify and sequence this region. In addition, the primer pair AB6/AB2, corresponding to nucleotides 281–300 and 870–889, was used to amplify the A/B region of the ER mRNA (exons 1 and 2) in these cell lines. To the 5′ end of each primer, two additional nucleotides were added to facilitate cutting at an introduced *Eco*RI restriction site. Reverse transcriptase–polymerase chain reaction products were gel purified and then cloned into pGEM7zf(+) vectors (Promega, Madison, WI, USA). Double-stranded plasmid DNAs containing the cDNA inserts were alkali denatured (Chen and Seeburg, 1985) and both strands were sequenced (Sequenase version 2.0, United States Biochemicals, Cleveland, OH, USA) using SP6 and T7 promoter primers (Sanger *et al.*, 1977), and then compared with those sequences reported in the Genetic sequence Data Bank (EMBL/GenBank).

### *RNAse protection analysis*

Thirty micrograms of total RNA from each ER-positive cell line (MCF-7, ZR-75-1, MDA-MB-134 and MDA-MB-361) and 60 μg of RNA from each ER-negative cell line (BT-20, T47Dco, BT-474, MDA-MB-330, MDA-MB-435S, MDA-MB-453, MDA-MB-231 and Hs578t) was hybridised to a $^{32}$P-labelled antisense cRNA ER variant probe lacking either exon 5 (Δ5) or exon 7 (Δ7). A [$^{32}$P]-labelled antisense cRNA probe generated from the cDNA for the constitutively expressed 36B4 gene product was utilised as a loading control. The Δ5 probe was generated by transcribing the ER variant cDNA clone containing portions of exons 4 and 6 (nucleotides 1142–1580) (Castles *et al.*, 1993), but with exon 5 (nucleotides 1389–1527) deleted. The Δ7 probe was generated using a cDNA clone containing portions of exons 6 and 8 (nucleotides 1542–2031) but with exon 7 (nucleotides 1662–1845) deleted. Radiolabelled variant ER antisense cRNA probe was generated using a Riboprobe Gemini II Core System (Promega) and purified by polyacrylamide gel electrophoresis. Hybridisation of $1\times10^6$ c.p.m. of labelled probe with sample RNA and subsequent digestion with RNAses A and $T_1$ were carried out using an RPA kit II (Ambion, Austin, TX, USA). The samples were heated at 85°C for 5 min, loaded onto a 6% polyacrylamide/8 M urea gel and electrophoresed at 1200 V for 4 h. Gels were dried and exposed to Kodak XAR film at −70°C for 18–48 h.

The numbers generated by densitometric analysis were corrected to account for the molar differences in size between the fully protected variant fragments and the partially protected wild-type fragment. The overall amount of radioactivity (probe) bound to the partially protected wild-type fragment identified in Figure 3 was approximately 20% less than that bound to the full-length protected fragment of the Δ5 variant transcript. Similarly, the numbers generated by densitometric analysis of the Δ7 assays were corrected to account for the molar differences in size between the two protected fragments. The partially protected wild-type fragment contained approximately 40% less radioactivity than the full-length protected fragment of the Δ7 variant transcript.

### Results

### *RT–PCR amplification, cloning and sequence analysis*

Total cellular RNA from the ER-positive MCF-7 and MDA-MB-361 cell lines and the ER-negative MDA-MB-330 and MDA-MB-231 cell lines was reverse transcribed and cDNA fragments from nucleotides 1142–1580 of the ER mRNA amplified by PCR using the primers HB10 and HB11. Amplified products were then separated on a 5% polyacrylamide gel. Similar to what has been previously demonstrated in some primary breast tumours (Fuqua *et al.*, 1991), all of these breast tumour cell lines except the MDA-MB-231 line expressed two amplified products: a larger 438 bp product which corresponds in size to the expected wild-type ER product and a smaller 300 bp variant band (Figure 1a). Dideoxynucleotide sequence analysis of the larger fragment from MCF-7, MDA-MB-361, BT-20 and MDA-MB-330 cells confirmed sequences established for the human ER (Ponglikitmongkol *et al.*, 1988), while the smaller variant fragment was found to contain wild-type sequences for exons 4 and 6 with exon 5 deleted (Castles *et al.*, 1993).

Reverse transcriptase–polymerase chain reaction amplification of total RNA from the four cell lines mentioned above was also performed using the primers HB18 and ERY6 to generate cDNA fragments from nucleotides 1553–2042 of the ER mRNA (Figure 1b). The MCF-7, MDA-MB-361 and MDA-MB-330 cell lines expressed two amplified products: a larger 489 bp product which correlated with the expected size of the wild-type ER product and a smaller 305 bp variant transcript. Clones from each of these cell lines containing the 489 bp product were sequenced and shown to correspond to the wild-type sequence for the ER. A representative sequence from the MDA-MB-330 cell line is shown in Figure 2. Sequence analysis of the cloned 305 bp product from MDA-MB-330 (Figure 2) as well as other cell lines (MCF-7, BT-20 and MDA-MB-361) revealed wild-type sequences for exons 6 and 8 with exon 7 deleted. Complete sequence analysis of both the Δ5 and Δ7 variants indicates that these deletions correspond to known intron/exon boundaries. The MDA-MB-231 cell line failed to express any ER mRNA transcripts.

**Figure 1** RT-PCR analysis of ER mRNA transcripts from human breast tumour cell lines. Total RNA from MCF-7, MDA-MB-361, MDA-MB-330 and MDA-MB-231 human breast tumour cell lines was reverse transcribed and amplified using the primer sets Hb10-Hb11 (**a**) and Hb18-ERY6 (**b**). Amplified products were run on a 5% polyacrylamide gel, stained with ethidium bromide and photographed. Arrows indicate exon deletion variant or wild-type ER cDNA generated from each cell line. All cell lines, with the exception of the MDA-MB-231 cell line, expressed Δ5 (300 bp) (**a**), Δ7 (305 bp) (**b**) and wild-type ER transcripts (438 and 489 bp respectively), as shown by the presence of amplified products. The molecular weight marker used in this gel is Phi DNA digested with *Hae*III. The numbers inside the cDNA box correspond to the exons of the ER cDNA.

**Figure 2** Sequence analysis of ER mRNA variants lacking exon 7 from BT-20 human breast cancer cells. Total RNA from the MCF-7, BT-20, MDA-MB-361, MDA-MB-330 and MDA-MB-231 cell lines was reverse transcribed and the cDNA amplified by PCR. Variant and wild-type PCR-derived cDNAs were cloned into pGEM-7zf(+) vectors and subjected to dideoxysequence analysis. Arrows indicate exon boundaries. The MCF-7, MDA-MB-361, MDA-MB-330 and BT-20 cell lines expressed the wild-type ER mRNA coexpressed with the exon 5 (not shown) and exon 7 deletion variants.

**Figure 3** RNAse protection analysis of ER mRNA from a panel of human breast cancer cell lines using an exon 5 deletion antisense cRNA. Fifty micrograms of total RNA from these cell lines was hybridised with an antisense cRNA probe prepared from a PCR-derived variant ER cDNA with exon 5 deleted. The ER-positive MCF-7, ZR-75-1, MDA-MB-361 and MDA-MB-134 cell lines, as well as the ER-negative (by ligand-binding analysis) BT-474, T47Dco, MDA-MB-330 and BT-20 cells, expressed both the fully protected Δ5 transcript (300 nt fragment) and the partially protected wild-type ER mRNA fragment (245 nt). No ER transcripts were identified in the ER-negative MDA-MB-435S, MDA-MB-453, MDA-MB-231 and Hs578t cell lines. The predominant transcript varied among the panel of cell lines examined. The molecular weight standard is pBR322 DNA digested with the restriction endonuclease *MspI*. The 36B4 cRNA was used to monitor RNA loading.

### *Detection and quantitation of ER mRNA transcripts in a panel of breast tumour cell lines using RNAse protection analysis*

In order to determine if the Δ5 and Δ7 ER mRNA variants are expressed in a panel of human breast tumour cell lines and to compare the relative level of expression of these variants with that of the wild-type ER, RNAse protection analysis was performed using antisense cRNA probes generated from the cDNAs containing the exon deletions as described above. A fully protected 300 nt fragment corresponding to the Δ5 variant and a partially protected 246 nt fragment corresponding to the wild-type ER mRNA were detected in the ER-positive MCF-7, ZR-75-1, MDA-MB-361 and MDA-MB-134 and the ER-negative MDA-MB-330, BT-474, T47Dco and BT-20 cell lines (Figure 3). The true ER-negative MDA-MB-435S, MDA-MB-453, MDA-MB-231 and Hs578t cell lines did not express any ER transcripts. As seen in Figure 4, each cell line which expressed a Δ5 variant ER transcript coexpressed some amount of wild-type message as well, although the expression ratio of variant to wild-type transcript differed among the various cell lines examined. In addition to the wild-type and Δ5 transcripts, the MDA-MB-231 cells also contained a novel partially protected fragment at approximately 287 nt. The exact make-up of this fragment has not been completely determined at present. In order to see clearly wild-type and variant transcripts in the ER-negative cell lines (e.g. BT-20 and MDA-MB-330), longer film exposure times were used. This resulted in all transcripts from the ER-positive cell lines being overexposed in Figure 3. Densitometric analysis was, however, performed on films that were not overexposed.

A fully protected 306 nt fragment corresponding to the Δ7 variant and a partially protected 185 nt fragment corresponding to the wild-type and other ER mRNA transcripts (e.g. Δ5) were detected in the ER-positive MCF-7, ZR-75-1, MDA-MB-361 and MDA-MB-134 cell lines (Figure 4). While many of the ER-negative cell lines (MDA-MB-435S, MDA-MB-453, MDA-MB-231 and Hs578t) failed to express any detectable levels of ER mRNA, some of the cell lines previously reported as ER negative (based on ligand-binding

**Figure 4** RNAse protection analysis of ER mRNA from a panel of human breast cancer cell lines using the exon 7 deletion antisense cRNA. Fifty micrograms of total RNA from these cell lines was hybridised with an antisense cRNA probe prepared from a PCR-derived variant ER cDNA with exon 7 deleted. The ER-positive MCF-7, ZR-75-1, MDA-MB-361 and MDA-MB-134 cell lines as well as the ER-negative (by ligand-binding analysis) BT-474, T47Dco, MDA-MB-330 and BT-20 cells expressed both a fully protected Δ7 transcript (305 nt fragment) and a partially protected wild-type fragment (184 nt). The molecular weight standard is pBR322 DNA digested with *MspI*. The 36B4 cRNA was used to monitor RNA loading.

**Figure 5** Composite densitometric analysis of RNAse protection assays. Following densitometric scanning of RPA autoradiographs, the relative expression of wild-type and variant ER mRNA species was compiled for the panel of human breast cancer cell lines. The predominant ER transcript varied among the cell lines, with some cell lines (e.g. MCF-7) expressing more wild-type ER mRNA and other cell lines (e.g. MDA-MB-361) expressing more Δ5 variant. The Δ7 variant transcript was constitutively expressed at low levels in all cell lines except the MDA-MB-134 and BT-20 cell lines.

analysis) did express both Δ7 variant and wild-type ER transcripts. These include the MDA-MB-330, T47Dco, BT-20 and BT-474 cell lines. A complete densitometric analysis of each RNAse protection assay is graphically represented as the combined expresson of both the Δ5 and Δ7 variants and the wild-type ER mRNA transcripts (Figure 5). Densitometric analysis of autoradiographs demonstrated that the Δ7 variant transcript appeared to be less abundant than either the Δ5 variant or the wild-type transcript in cell lines expressing detectable levels of ER mRNA. The one exception to this was the BT-20 cell line, which expressed slightly more Δ7 variant than wild-type.

Further analysis revealed that differences in the expression levels of the ER variants among the cell lines were more pronounced for the ratio of the Δ5 variant to wild-type (Figure 5) than for Δ7 to wild type. For example, in the MCF-7 cell line (Figure 5, Table I), the wild-type ER mRNA accounted for over 57% of the ER transcripts, while the Δ5 variant accounted for 39% and the Δ7 variant less than 4% of the total ER mRNA transcripts. In the weakly ER-positive MDA-MB-361 cells, the Δ5 variant accounted for 51% of the ER mRNA transcripts, the wild-type accounted for 34% and the Δ7 variant 17% of the total ER mRNA species (Table I). Conversely, in BT-20 cells, which are classified as ER negative based on ligand-binding analysis, the Δ5 variant transcript made up over 68% of the total ER mRNA transcripts, while the Δ7 variant accounted for 24% and the wild-type approximately 8% of the total ER transcripts. Like the BT-20 cells the MDA-MB-134 cell line was the only ER-positive cell line that expressed elevated levels of the Δ7 transcript (25%). This assay methodology (RNAse protection analysis) generates different molar ratios (different sized radiolabelled protected fragments) between the fully protected variant and the partially protected wild-type transcript. Since the smaller protected fragment represents less radioactive RNA than the larger variant fragment, true expression differences between variant and wild-type transcripts are not clearly evident upon visual inspection of autoradiographs. Thus, for accurate measurement of transcript ratios, mathematical manipulations were performed on the densitometric data to correct for the molar differences in fragment size. In addition, since the smaller protected fragments in both Δ5 and Δ7 assays contain wild-type ER mRNA as well as one of the variant transcripts (depending on which variant probe is used), further adjustments in the expression levels were made by subtracting Δ5 or Δ7 levels from the smaller fragment in the parallel assay with the other probe.

**Table I** Average per cent composition of total ER mRNA from a panel of breast cancer cell lines

| *Cell line* | *Wild-type (%)* | *Δ5 variant (%)* | *Δ7 variant (%)* |
|---|---|---|---|
| MCF-7(M) | 57 | 39 | 4 |
| ZR-75-1 | 41 | 47 | 12 |
| MDA-MB-361 | 41 | 51 | 7 |
| MDA-MB-134 | 34 | 41 | 25 |
| BT-474 | 41 | 44 | 15 |
| T47Dco | 39 | 47 | 14 |
| BT-20 | 8 | 68 | 24 |
| MDA-MB-330 | 27 | 58 | 15 |
| MDA-MB-435S | – | – | – |
| MDA-MB 453 | – | – | – |
| MDA-MB-231 | – | – | – |
| Hs578t | – | – | – |

Note that in some cell lines, such as MCF-7, the wild type was the predominant transcript, while in other cell lines, such as MDA-MB-361 and BT-20, the Δ5 deletion variant was the predominant transcript. Samples were normalised to the loading control. These data are based on the mean of three assays ± s.e.

### Discussion

We report here that a number of human breast tumour cell lines express ER mRNA variants which contain precise deletions of exons 5 and/or 7. Several ER-positive cell lines as well as some ER-negative cell lines were found to contain varying levels of both wild-type and the two ER variant transcripts. These cell lines include the ER-positive MCF-7, ZR-75-1, MDA-MB-134 and MDA-MB-361 as well as the ER-negative BT-20, MDA-MB-330, BT-474 and T47Dco cell lines. Some ER-negative cell lines, including MDA-MB-231, MDA-MB-435s, MDA-MB-453 and Hs578t, failed to express any detectable levels of ER and mRNA.

We have previously identified and characterised the Δ5 variant ER transcript in the ER-positive MCF-7 and ER-negative BT-20 cell lines (Fuqua *et al.*, 1991; Castles *et al.*, 1993). This variant transcript encodes a transcriptionally active, dominant-positive variant ER protein. According to our RNAse protection analyses, this variant transcript is the predominant ER mRNA species expressed in the ER-positive MDA-MB-361, MDA-MB-134 and ER-negative BT-20 and MDA-MB-330 cell lines, accounting for 51%, 68%, 41% and 58%, respectively, of the total ER mRNA. We have also previously identified (Fuqua *et al.*, 1992*a*) in breast tumour specimens a Δ7 ER mRNA variant that encodes a dominant-negative receptor which interferes with the ability of the wild-type receptor to activate gene transcription in a yeast reporter assay system. This particular variant ER transcript, along with other variants lacking exons 2 or 3, has been previously identified in the ER-positive T47D cell line (Wang and Miksicek, 1992), as well as in conjunction with a deletion of exon 4 in MCF-7 cells (Koehorst *et al.*, 1993).

The coexpression of ER mRNA variants with the wild type is in agreement with earlier reports examining breast tumour specimens (Fuqua *et al.*, 1991, 1992*a*; Zhang *et al.*, 1993) and the T47D, ZR-75-1, and MCF-7 cell lines (Wang and Miksicek, 1992; Koehorst *et al.*, 1993). However, based on RNAse protection analysis, the ratio of expression of the Δ5 and Δ7 mRNA variants to each other as well as to the wild-type ER transcript differed among the cell lines examined. This can also be observed in panels of ER-positive/PgR-positive and ER-positive/PgR-negative breast tumours (SAW Fuqua, unpublished data). In cell lines such as the ER-positive MCF-7 cell line (Figure 5, Table I), the wild-type ER transcript accounted for over 57% of the total ER transcripts, while the Δ5 transcript made up 39% and the Δ7 variant less than 4%. In the ER-positive MDA-MB-134 cell line, the Δ5 ER variant accounted for 41% of the ER mRNA transcripts, with the wild-type making up 34% and the Δ7 variant 25%. In the BT-474 cell line, the Δ5 variant and wild-type ER mRNA transcripts were expressed at nearly equivalent levels, while only minimal levels of the Δ7 transcript were expressed. This cell line, classified as ER negative by Lasfargues *et al.* (1978) based on ligand-binding analysis, constitutively expresses high levels of PgR. However, a recent report by Hall *et al.* (1990) indicates that this cell line does, in fact, express an ER transcript.

In the T47Dco cell line, which is also ER negative/PgR positive (Horwitz, 1982), the wild-type message is not the predominant ER transcript, making up only 39% of the total amount of ER mRNA transcripts, with the Δ5 variant accounting for 47%. However, in this cell line, ER transcripts are expressed at levels well below the ER-positive cell lines. In the ER-negative BT-20 cell line, the Δ5 variant made up over 68% of the ER mRNA transcripts, with the wild-type accounting for only 8% of the detectable ER transcripts. This was the only cell line examined which expressed levels of the Δ7 variant (24%) which were equivalent or slightly greater than the levels of the wild-type (8%) ER transcript. However, in all of these cell lines there did not appear to be a significant correlation between the level of expression of the Δ5 variant ER mRNA transcripts and oestrogen-binding activity as measured by ligand-binding analysis as demonstrated by the T47Dco ($ER^-$) and MCF-7 ($ER^+$) cell lines, which both express wild type as the predominant ER transcript. Conversely, two ER-positive cell lines (MDA-MB-134 and MDA-MB-361) and two ER-negative cell lines (BT-20 and MDA-MB-330) expressed predominantly the Δ5 ER variant transcript. All cell lines expressing ER transcripts but classified as ER negative by ligand-binding analysis expressed significantly lower levels of all ER transcripts than did their ER-positive counterparts. It should again be pointed out that the expression ratios of the ER mRNA variants to each other and to the wild-type transcript, as well as the per cent expression of each ER mRNA species, were based solely upon the RNAse protection analysis. These results differ somewhat from the recent observations of Zhang *et al.* (1993), who used this same technique to examine the expression of the Δ5 variant transcript in the MCF-7 and ZR-75-1 breast tumour cell lines. While the expression ratios of Δ5 to wild type were similar for MCF-7 cells in both studies, the ZR-75-1 cells in our study had an expression ratio approaching 1:1; in contrast Zhang *et al.* reported a ratio of 0.5:1. These differences may be the result of inter-assay variation. However, we have recently reported that different stocks of the MCF-7 cell line express considerably different ratios of Δ5 to wild type transcripts, and that the mitogenic effects of 17β-oestradiol in the various MCF-7 stocks is significantly correlated with the expression ratio of wild-type to variant ER mRNA (Klotz *et al.*, 1994). Thus, the differential expression of variant to wild-type ER mRNA may be related to the oestrogen responsiveness of the cells. It is apparent that the Δ5 variant is expressed at higher levels than the Δ7 variant in all cell lines examined, suggesting that these two deletions do not reside on the same ER mRNA transcript, but may be the result of alternative splicing and the formation of separate transcripts.

Although we have identified Δ5 and Δ7 ER mRNA variants in several breast tumour cell lines, as yet there is no clear evidence that ER variant proteins serve a specific function in either normal or cancerous cells *in vivo*. We have, however, identified in MCF-7 and BT-20 breast tumour cells immunoreactive proteins corresponding in size to the wild-type ER (65 kDa) and the Δ5 ER variant (42 kDa (Castles *et al.*, 1993). In the current studies we have not attempted to quantitate the protein levels since such studies would involve immunoprecipitation and Western blot analysis using the H226 ER monoclonal antibody, whose production has been discontinued by our supplier Abbott Laboratories (Abbott, IL, USA). In a yeast expression system, the Δ5 ER variant protein acts in a dominant-positive manner to activate constitutively transcription of an ER-regulated gene construct, while the Δ7 ER variant exhibits dominant-negative activity, interfering with the ability of the wild-type ER to initiate gene transcription (Fuqua *et al.*, 1991, 1992*a*; Castles *et al.*, 1993). Both the Δ5 and Δ7 variant proteins when expressed in yeast are truncated forms of the ER, with each variant missing a portion of the hormone-binding domain as well as the dimerisation and AF-2 domains located in the E region of the ER (Fawell *et al.*, 1990). Since the Δ5 variant is the predominant ER mRNA isoform in the BT-20 cell line (Castles *et al.*, 1993), which is ER negative by ligand-binding analysis, and since Fuqua *et al.* (1992*b*) have shown that, when expressed in MCF-7 cells, this variant is able to confer hormone independence and tamoxifen resistance, it is possible that expression of this variant may contribute to the hormone indepenent proliferation of the BT-20 and MDA-MB-330 cells.

The Δ7 ER variant has been shown, by our group, to act in a dominant-negative manner, apparently dimerising with the wild-type receptor to form an inactive heterodimer (Fuqua *et al.*, 1992*a*). This variant is expressed at higher levels in the MDA-MB-134 cell line than in other ER-positive cell lines. It is interesting to note that this cell line, although ER positive by ligand binding, is not induced to express PgR in response to oestrogen stimulation (data not shown) (Reiner *et al.*, 1986). In addition, this cell line also expresses, as determined by RNAse protection analysis, a partially protected fragment of intermediate size between the wild-type and the Δ5 transcript. The exact nature of this partially protected fragment has not been fully characterised so far. Considering the high level of Δ7 expression in this cell line, it is tempting to speculate that elevated levels of this variant may play a role in the lack of PgR induction by oestradiol, although oestrogen-induced proliferation is not inhibited in these cells. The low-level constitutive expression of PgR in these cells might then be due to gene activation by the constitutively active truncated Δ5 ER variant. This variant would not be expected to dimerise with the Δ7 variant since it is truncated and missing its dimerisation domain. Further studies to characterise these variant ERs, such as analysis of DNA-binding properties, will be needed to determine the true clinical significance of such variants. The coexpression of the Δ5 and Δ7 variant transcripts with the wild-type message in both ER-positive and ER-negative breast cancer cell lines may simply be a reflection of the cancerous state of these cells. However, this is probable not the case since these and other ER mRNA variants have been identified in normal/non-malignant tissues such as the uterus and brain (Koehorst *et al.*, 1993). Thus, they appear to be naturally occurring splicing variants which may play a role in the development or normal function of some oestrogen-responsive tissues. It is possible that the overexpression of such variants in relation to the wild-type ER might be related to the development of a malignant phenotype or to hormone resistance in some breast tumours. Given the potential biological action of these ER variants, it is probably that their differential expression may be an important factor in modulating the overall oestrogen responsiveness of various tissues as well as the expression of oestrogen responsive genes.

**Acknowledgements**
This work was supported by American Cancer Society Grant No. BE-72794 to SMH and Louisiana Cancer and Lung Trust Fund Grant to SMH. A special note of thanks is due to Marty Bitner for the typing of this manuscript.

### References

BENNER SE, CLARK GM AND MCGUIRE WL. (1988). Review: steroid receptors, cellular kinetics, and lymph node status as prognostic factors in breast cancer. *Am. J. Med. Sci.*, **296**, 59-66.

CASTLES CG, KLOTZ DM, FUQUA SAW AND HILL SM. (1993). Expression of a constitutively active estrogen receptor variant in the estrogen-receptor-negative BT-20 breast cancer cell line. *Cancer Res.*, **53**, 5934-5939.

CHEN EY AND SEEBURG PH. (1985). Supercoil sequencing: a fast and simple method for sequencing plasmid DNA. *DNA*, **4**, 165-170.

CHOMCZYNSKI P. AND SAACHI N. (1987). Single-step method of RNA isolation by acid guanidinium thiocyanate-phenol-chloroform extraction. *Ann. Biochem.*, **162**, 156-159.

EDWARDS DP, CHAMNESS GC AND MCGUIRE WL. (1979). Estrogen and progesterone receptors in breast cancer. *Biochim. Biophys. Acta*, **560**, 457-486.

EVANS RM. (1988). The steroid and thyroid hormone receptor superfamily. *Science*, **240**, 889-895.

FAWELL SE, LEES JA, WHITE R AND PARKER MG. (1990). Characterization and co-localization of steroid binding and dimerization activities in the mouse estrogen receptor. *Cell*, **60**, 953-962.

FUQUA SAW, FITZGERALD SD, CHAMNESS GC, TANDON AK, MCDONNELL DP, NAWAZ A, O'MALLEY BW AND MCGUIRE WL. (1991). Variant human breast tumour estrogen receptor with constitutive transcriptional activity. *Cancer Res.*, **51**, 105-109.

FUQUA SAW, FITZGERALD SD, ALLRED CD, ELLEDGE RE, NAWAZ Z, MCDONNELL DP, O'MALLEY BW AND MCGUIRE WL. (1992*a*). Inhibition of estrogen receptor action by a naturally occurring variant in human breast tumors. *Cancer Res.*, **52**, 483-486.

FUQUA SAW, ALLRED DC, NAWAZ Z, GREENE GL AND MCGUIRE WL. (1992*b*). A dominant-positive variant estrogen receptor confers resistance to endocrine therapy in human breast cancer cells (abstract 1088). *Proceedings of the 74th Annual Meeting of the Endocrine Society*, San Antonio, TX, p. 323. Endocrine Society Press: Bethesda, MD.

FUQUA SAW, ALLRED DC, ELLEDGE RM, KRIEG SL, BENEDIX, MG, NAWAZ Z, O'MALLEY BW, GREENE GL AND MCGUIRE WL. (1993). The ER-positive/PgR-negative breast cancer phenotype is not associated with mutations within the DNA-binding domain. *Breast Cancer Res. Treat.*, **26**, 191-202.

GRAHAM ML, KRETT NL, MILLER LA, LESLIE KK, GORDON DF, WOOD WM, WEI LL AND HORWITZ KB. (1990). $T47D_{CO}$ cells, genetically unstable and containing estrogen receptor mutations, are a model for the progression of breast cancer to hormone resistance. *Cancer Res.*, **50**, 6208-6217.

GREEN S AND CHAMBON P. (1991). The oestrogen receptor: from perception to mechanism. In *Nuclear Hormone Receptors*, Parker MG (ed.) pp. 15-38, Academic Press: San Diego.

GREENE GL, GILNA P AND WATERFIELD M. (1986). Sequence and expression of human estrogen receptor complementary DNA. *Science*, **231**, 1150-1154.

HALL RM, LEE SSL, ALEXANDER IE, SHINE J, CLARKE CL AND SUTHERLAND RL. (1990). Steroid hormone receptor gene expression in human breast cancer cell: Inverse relationship between oestrogen and glucocorticoid receptor messenger RNA levels. *Int. J. Cancer*, **46**, 1081-1087.

HORWITZ KB, MOCKUS MB AND LESSEY BA. (1982). Variant T47D human breast cancer cells with high progesterone-receptor levels despite estrogen and antiestrogen resistance. *Cell*, **28**, 633-642.

KLOTZ DM, CASTLES CG AND HILL SM. (1994). Differential expression of wild type and variant ER mRNA by clones of MCF-7 breast cancer cells may account for differences in estrogen-responsiveness (abstract 1070). *Proceedings of the 76th Annual Meeting of the Endocrine Society*, Anaheim, CA, p. 468. Endocrine Society Press: Bethesda MD.

KOEHORST SGA, JACOBS HM, THIJSSEN HHH AND BLANKENSTEIN MA. (1993). Wild-type and alternatively spliced estrogen receptor messenger RNA in human meningioma tissue and MCF-7 breast cancer cells. *J. Steroid Biochem. Mol. Biol.*, **45**, 227-233.

KUMAR V, GREEN S, STACK G, BERRY M, JIN JR AND CHAMBON P. (1987). Functional domains of the human estrogen receptor. *Cell*, **51**, 941-951.

LASFARGUES EY, COUTINHO WG AND REDFIELD ES. (1978). Isolation of two human tumor epithelial cell lines from solid breast carcinomas. *J. Natl Cancer Inst.*, **61**, 967-973.

MCGUIRE WL, CARBONE PP, SEARS ME AND ESCHERT GC. (1975). Estrogen receptors in human breast cancer: an overview. In *Estrogen Receptors in Breast Cancer*, McGuire WL, Carbone PP and Voller EP (eds) pp. 1-7, Raven Press: New York.

MCGUIRE WL, CHAMNESS GC AND FUQUA SAW. (1992). Abnormal estrogen receptor in clinical breast cancer. *J. Steroid Biochem. Mol. Biol.*, **43**, 243-247.

PFEFFER U, FECAROTTA E, CASTAAGNETTA L AND VIDALI G. (1993). Estrogen receptor variant messenger mRNA lacking exon 4 in estrogen-responsive human breast cancer cell lines. *Cancer Res.*, **53**, 741-743.

PONGLIKITMONGKOL M, GREEN S AND CHAMBON P. (1988). Genomic organization of the human estrogen receptor. *EMBO J.*, **7**, 3385-3388.

REINER GCA AND KATZENELLENBOGEN BS. (1986). Characterization of estrogen and progesterone receptors and the dissociated regulation of growth and progesterone receptor stimulation by estrogen in MDA-MB-134 human breast cancer cells. *Cancer Res.*, **46**, 1124-1131.

SANGER F, NICKLEN S AND COULSON AR. (1977). DNA sequencing with chain-terminating inhibitors. *Proc. Natl Acad. Sci. USA*, **74**, 5463-5467.

WALTER P, GREEN S, GREENE G, KRUST A, BORNERT A, JELTSCH JM, STAUB A, JENSEN E, SCRACE G, WATERFIELD M AND CHAMBON P. (1985). Cloning of the human estrogen receptor cDNA. *Proc. Natl Acad. Sci. USA*, **82**, 7889-7893.

WANG Y AND MIKSICEK RJ. (1991). Identification of a dominant negative form of the human estrogen receptor. *Mol. Endocrinol.*, **5**, 1707-1715.

ZHANG QT, BORG A AND FUQUA SAW. (1993). An exon 5 deletion variant of the estrogen receptor frequently coexpressed with wild-type estrogen receptor in human breast cancer. *Cancer Res.*, **53**, 5882-5884.